# Validation of the coping self-efficacy scale: Vietnamese version for adolescents

**DOI:** 10.1186/s40359-022-00770-3

**Published:** 2022-03-09

**Authors:** Thach Tran, Nga La, Huong Nguyen, Ian Shochet, Nga Nguyen, Astrid Wurfl, Jayne Orr, Hau Nguyen, Ruby Stocker, Jane Fisher

**Affiliations:** 1 Global and Women’s Health, Public Health and Preventive Medicine, Monash University, Level 4 - 553 St Kilda Road, Melbourne, VIC 3004 Australia; 2 Hanoi University of Public Health, Hanoi, Vietnam; 3 School of Psychology and Counselling, Queensland University of Technology, Brisbane, Australia

**Keywords:** Construct validity, Coping self-efficacy, Stress, Adolescents, Vietnam

## Abstract

**Background:**

This study aimed to examine the construct validity of the Coping Self-Efficacy Scale-Vietnamese Version (CSES-V) among Vietnamese adolescents.

**Methods:**

This study selected Grade 10 students from eight schools in Hanoi using a multiple-stage sampling method. Multiple aspects of the construct validity were examined including: factorial structure (evaluated using exploratory factor analysis); internal consistency (tested using Cronbach’s alpha coefficient); measurement invariance between male and female participants and longitudinal measurement invariance (tested by employing multiple group confirmatory factor analysis) and external aspect (tested using Pearson’s correlation coefficients between CSES-V and the Depression Anxiety and Stress Subscales of Depression (DASS21-D), Anxiety (DASS21-A), and Stress (DASS21-S) and a measure of mental well-being, Mental Health Continuum Short Form (MHC-SF)).

**Results:**

A total of 1082 adolescents (aged 14–16 years) was included in this study. Data supported a three-factor structure (comprising 24 items) that explained 97.6% of the total variance of the CSES-V. Cronbach’s alpha coefficients of all three factors were acceptable. All levels of measurement invariance between male and female participants and longitudinal measurement invariance were well-supported. The three factors of the CSES-V were positively correlated with MHC-SF and were negatively correlated with the DASS21 subscales at a low or moderate level, supporting the external aspect of the construct validity.

**Conclusions:**

CSES-V is recommended to assess coping self-efficacy among Vietnamese adolescents who are attending school.

**Supplementary Information:**

The online version contains supplementary material available at 10.1186/s40359-022-00770-3.

## Background

Stress is a person-environment relationship that arises when a person perceives it as exceeding their ability to cope with the threats or demands being made on them [1, 2]. There are many life events or situations that can become stressors; for example, interpersonal conflict, death of loved ones, illness, heavy workload or excessive responsibility. Stress is common in every stage of life [3]. Stress, especially if severe and prolonged, can be a triggering factor for many diseases and pathological conditions including cognitive and mental health problems [4–6].

Lazarus & Folkman’s Stress and Coping Theory [1] defines coping as the thoughts or actions employed to manage stressful situations. Developing a coping strategy for each stressful event is the result of an individual cognitive evaluative process (appraisal) of the circumstance. Two stages are involved: (1) the prediction of adverse outcomes (primary appraisal), and (2) consideration of different ways to respond (secondary appraisal). Coping strategies can be categorised into problem-focused and emotion-focused coping. Problem-focused coping concentrates on changing the stressor itself and its physical impact. Emotion-focused coping centres around managing emotional responses to stressful events. However, some coping responses do not fit completely into either category. For instance, seeking social support can be a problem-focused effort (change the situation) or an emotion-focused action (soothe the distressing emotion). When the coping strategies fit the stressful events/situations well, people can diminish the influence of stressful experiences, and in turn reduce immediate and future psychological and physical health impacts [7].

Coping with stress self-efficacy is a construct that was recently formulated by integrating the Lazarus & Folkman’s Stress and Coping Theory [1] and Bandura’s Self-efficacy Theory [8]. In general, perceived self-efficacy is the belief an individual has about their ability to adequately perform a specific behaviour. Specific self-efficacy for coping with stress is an individual’s subjective judgement about their own ability to handle stressful situations effectively [9]. Self-efficacy for coping with stress affects both appraisal stages in the Stress and Coping Theory. First, an individual’s degree of belief that they can solve the problem or regulating their emotions determines the adverse outcome they predict. Second, perception of self-efficacy plays a crucial role in the choice and implementation of coping strategies. Individuals will choose, organise and carry out actions that they believe are useful and effective for dealing with the situation. Therefore, high self-efficacy for coping with stress can prevent or reduce the stress as well as its health impacts.

Research interest in stress and coping became widespread in 1970s and 1980s [10], leading to the development of a number of instruments to assess coping with stress (for instance, the Miller Behavioral Style Scale [11]; the Ways of Coping Questionnaire [12]; the COPE Inventory [13]; the Coping Strategy Indicator [14]; the Mainz Coping Inventory [15]; and the Coping Inventory for Stressful Situations [16]). However, all of these instruments assess coping strategies per se, rather than self-efficacy for coping. Chesney and colleagues, in collaboration with Dr. Albert Bandura from Stanford University, who postulated a self-efficacy theory [8], developed the Coping Self-Efficacy Scale (CSES), one of very few scales to measure perceived self-efficacy for coping with challenges and threats [7]. A total of 26 behaviours is asked about in the CSES and grouped into three categories of coping strategies: problem-focused (12 items), emotion-focused (9 items), and get support from friends and family (5 items). Chesney et al. [7] was the first to empirically examine the construct validity of the CSES. The 3-factor structure (i.e., problem-focused, emotion-focused, and getting social support) yields strong internal consistency and test–retest reliability supported by the data. Concurrent validity (correlations between the CSES and measures of psychological distress and well-being, ways of coping, and social support) and predictive validity (change scores in using problem- and emotion-focused coping skills were predictive of reduced psychological distress and increased psychological well-being over time) were established. A shortened version with 13 items was proposed. However, Chesney et al. suggested using the full version of 26 items to recheck the construct validity, because the first validation study included a sample of participants who were homosexual men infected with HIV and diagnosed with depressed mood, and were thus not representative of the general population.

There were some other attempts to validate the CSES [17–20]. Among those, the most outstanding study was conducted by Colodro et al. [18] with a community-based sample of 182 adults from 18 to 66 years of age in the UK. Overall, the findings in the Chesney et al.’s study [7] including the factorial structure, concurrent validity and predictive validity of the full version of CSES were confirmed with data from the community-based sample in Colodro et al.’s study. However, three of the 26 items with lowest loadings were suggested as not ‘sufficiently suitable for inclusion in the scale’. Another validation study, conducted in South Africa by van Wyk [20], included a convenience sample of 2214 people aged from 16 to 46 years. Chesney’s original factorial structure of the CSES fitted van Wyk’s study data well. Van Wyk’s data also support high internal consistency (Cronbach alpha of 0.87) and good criterion-related validity of the CSES. Cunningham et al. [17] validated the CSES among a clinical sample of military service members receiving mental health or substance abuse treatment in the USA. The original three-factor model was supported in Cunningham et al.’s study. Finally, Tol et al. [19] validated an Iranian version of the CSES for use among people with type 2 diabetes mellitus. The original factorial structure of the CSES was not supported; instead two items were omitted and a four-factor model was found to fit the data well.

Common mental health problems, especially depression and anxiety, are prevalent worldwide at almost every stage of life including adolescence [21]. For many people with common mental health problems, the first onset occurs during adolescence [22, 23]. Mental health problems during this period are associated with higher risks of subsequent mental health problems in adulthood [24]. Therefore, public health interventions for adolescents’ mental health are urgently needed not only for current adolescents’ well-being but also for future adults’ quality of life and productivity.

Mental health problems among adolescents have been recognised in public health research in Vietnam for more than a decade. In 2011, Amstadter and colleagues [25] reported data from a large-scale community-based study that 9.1% of adolescents aged 11–18 years were considered to have a mental health problem. Recent studies found that up to 22.9% of adolescents experienced clinically significant symptoms of depression [26], 22.8% had clinically significant symptoms of anxiety [27], and 14.1% had suicidal thoughts [28]. Although common mental health problems including depression and anxiety are being recognised increasingly by policy makers, there are a lack of public health interventions to support adolescent mental health in Vietnam.

High coping self-efficacy is a protective factor for depression and anxiety disorders [29, 30]. Coping self-efficacy is increasingly recognised as being changeable through psycho-educational programs [31]. There have been a number of recent attempts to develop programs aiming to promote positive coping self-efficacy for addressing mental health problems [7]. An instrument to assess coping self-efficacy is necessary for these interventions and research. To our knowledge, there is no coping self-efficacy scale that has been validated for use among adolescents.

This study aimed to examine the construct validity of the CSES for use among high school students in Vietnam. The CSES was selected for several reasons: (1) it covers all major domains of coping strategies (i.e. problem-focused, emotion-focused, and help seeking), (2) it has 26 items, meaning it is not too brief and not too long, (3) the 11-point scale for each item means that the scale can provide detailed data, and (4) it evaluates the person’s confidence regarding implementing coping strategies, and changes in scale scores reflect changes in the individual’s confidence regarding their ability to cope. The CSES holds great promise for use in public health and research to inform effective interventions to help adolescents better handle both acute and chronic stress [7].

The objectives of this study were to evaluate the (1) factorial structure, (2) measurement invariance, (3) internal consistency, and (4) concurrent validity of a Vietnamese version of the CSES. We used data collected from an intervention study (hereafter called the *main study*) of a school-based psycho-educational program for adolescents’ mental health conducted in Hanoi, Vietnam [32]. We hypothesised that the data would support all aspects of construct validity of the CSES.

## Methods

### Settings

Vietnam is Southeast Asian country with a population of 96 million. The average national per capita income in 2019 was USD2,590, and Vietnam is classified as a lower-middle income country [33]. Children and adolescents account for a third of the population. Nationally, about 8.3% of school-age children (6–18 years old) are out of school [34]. Hanoi, the capital city, is one of the two largest cities in Vietnam. Of the 8 million people living in Hanoi, the population is split equally between those living in urban and rural areas.

### Participants

A multiple-stage sampling method was used in the *main study* to select the participants. In the first stage, two districts were randomly selected from a total of 12 urban districts and another two districts were randomly selected from a total of 18 rural districts in Hanoi. In the second stage, in each of the selected districts, two high schools were randomly selected and four grade 10 classes from each of the selected schools were randomly chosen. Finally, all students in the selected classes were eligible and invited to participate. An independent statistician conducted the selection process. A total of 1084 (552 controls and 532 interventions) adolescents aged 15–16 years participated in the *main study*.

All participants of the *main study* were eligible and included in this validation study.

### Procedures

In the *main study,* data were collected at baseline (at recruitment) and endline (about two months after recruitment) using a self-completed questionnaire at school during a usual 45-min class. In each session, two research assistants from the Hanoi University of Public Health (HUPH) gave instructions on how to complete the questionnaire and supervised the students to ensure the privacy and confidentiality. Students returned the questionnaire in a sealed envelope which was provided at the beginning of the session. Students who did not want to participate or did not have parental consent to participate were invited to go to do their homework at the school library (44 students, 3.9%).

### Measures

#### Coping self-efficacy scale-Vietnamese version

The Vietnamese Version of the original 26-item version of the Coping Self-Efficacy Scale (CSES-V) developed by Chesney and colleagues was used in this study [7]. For each item, students are asked to rate on an 11-point scale the extent to which they believe they could perform a behaviour when things aren’t going well, or when they are having problems (0 ‘cannot do at all’ to 10 ‘certain can do’). The translation into Vietnamese was performed using a standardised procedure (translate, culturally verify and back-translate) established and used in previous studies [35–37].

#### Depression anxiety and stress scales (DASS 21)

The symptoms of depression, anxiety and stress were assessed using the DASS 21 [38] which includes 21 items in three sub-scales (each has seven items): Depression (DASS21-D), Anxiety (DASS21-A), and Stress (DASS21-S). Each item has four short response options reflecting the severity of the symptom and scoring from 0 = “Did not apply to me at all” to 3 = “Applied to me very much, or most of the time”. Higher subscale scores indicate more symptoms of the mental health problem measured by the subscale. Evidence for the factorial structure and internal consistency of DASS 21 for use among Vietnamese adolescents has been established [39] (Cronbach alphas of 0.835 for the Depression, 0.737 for the Anxiety and 0.761 for the Stress subscale).

#### Mental health continuum short form (MHC-SF)

General mental well-being was assessed using the MHC-SF [40, 41]. The MHC-SF comprises 14 items and each item is scored from 0 = “Never” to 5 = “Every day”. All item scores are summed to yield a global well-being score from 0 to 70. Higher global well-being scores reflect better mental well-being. Ha et al. confirmed the construct validity of the MHC-SF for use in adolescents in Vietnam [42].

### Statistical analyses

In this study, we examined two aspects of construct validity of the CSES-V, namely structural and external validity [39].

#### Structural aspect

*The factorial structure* of the CSES-V was examined using exploratory factor analysis with principal factor extraction (free of distribution assumptions). The number of factors selected was decided based the scree plot (plot of the eigenvalues of factors) and meaningful factors. After the number of factors was determined, we use an oblique rotation (promax) to reach a simple structure if more than one factor was found. We omitted from the final version the items with factor loadings < 0.3, as they were interpreted as being not salient. For every item cross-loading into two or more factors, it was assigned to the factor with the highest factor loading value.

*Measurement invariance* (measuring the same construct(s) in the same way across the subgroups of participants) of the CSES-V was examined between male and female participants using multiple group confirmatory factor analysis (MGCFA) in three levels: configural; metric; and scalar invariance [43, 44]. The lowest level of measurement invariance, configural invariance, requires the number of factors and loading pattern to be the same across groups. The configural invariance holds if the overall MGCFA model fits the data well (the root mean square error of approximation (RMSEA) value of < 0.05, comparative fit index (CFI) > 0.95, and Tucker–Lewis index (TLI) > 0.95) [44, 45]. The second level of measurement invariance is the metric invariance level in which the factor loadings of the items of the instrument must be equivalent across groups. The fit of the metric model was compared with the fit of the configural model to assess metric invariance. The highest level of measurement invariance is scalar invariance, which requires the item intercepts to be equivalent across groups, in addition to the metric invariance. If the metric invariance is achieved, the fit of the scalar model is compared with the fit of the metric model to assess scalar invariance. We used the criteria: the decreases of CFI values of less than or equal to 0.01 and increases in RMSEA values of less than or equal to 0.015 from the compared model indicating that there is no difference between the models and invariance at that step is supported [46–48]. We did not use Chi-square tests to test model fit differences between models, because Chi-square tests are heavily influenced by the sample size [44].

*Longitudinal measurement invariance* (measuring the same construct in the same metric across time points) of the CSES-V was examined using the same statistical approach (MGCFA) in the three levels as in the examination of the measurement invariance between participants’ sexes.

*The internal consistency* of the scale was assessed using the Cronbach’s alpha coefficient. The coefficient > 0.7 indicates acceptable internal reliability [49].

#### External aspect

*Concurrent validity* (whether the CSES-V correlates with the measures of related constructs) was examined using Pearson’s correlation coefficients between CSES and DASS21-D, DASS21-A, DASS21-S, and MHC-SF scores. Stronger coping self-efficacy is negatively associated with depressive, anxiety and stress symptoms and positively associated with mental well-being [50–52]. It was expected that the CSES-V would be correlated with all measures at low or moderate levels (correlation coefficients around 0.3 to 0.5).

We used data collected from all participants at baseline in all analyses, except for the assessment of longitudinal measurement invariance. For the longitudinal measurement invariance, we used data collected at baseline and endline from participants of the control group only.

Several methods for treating missing data were used in this study. First, the cases with more than 20% of CSES-V data items missing were excluded. Second, missing data in the scales (CSES, DASS21-D, DASS21-A, DASS21-S, or MHC-SF) were imputed if a case had missing data for less than or equal to 20% of the number of items of that scale. Regression imputation was used; all other items of these scales and sociodemographic characteristics (school, sex, and age) were used as predictors to impute the missing data. Thirdly, the remaining missing data were treated using full information maximum likelihood estimation under missing at random assumption in the MGCFA. Finally, we used the pairwise deletion approach in other analyses. MGCFA were conducted in Mplus Version 7.4 [53]. All other analyses were carried out using Stata Version 16 [54].

The data, analytic methods (code) used in the analysis, and materials used to conduct the research will be made available to any researcher for purposes of reproducing the results or replicating the procedure on reasonable request to the corresponding author.

### Ethical considerations

This research was undertaken in accordance with the Australia’s National Statement on Ethical Conduct in Human Research and the Helsinki Declaration of 1975, as revised in 2008. This study has been approved by Monash University Human Research Ethics Committee (Certificate Number: 21455), Melbourne, Victoria, Australia; the Institutional Review Board of the Hanoi University of Public Health (488/2019/YTCC-HD3), Hanoi, Vietnam; and Queensland University of Technology's Office of Research Ethics and Integrity (2000000087). Written informed consent was obtained from a parent or guardian for participants under 16 years old.

## Results

### Samples

Among the 1084 students who participated in the *main study*, 13 (1.2%) had missing data in any CSES item at baseline. We excluded two cases (0.2%, one in control and one in intervention group) who had more than 20% CSES-V data items missing. There were 76 participants (7.0%) missing any data in items in the DASS scales, and 59 (5.4%) missing any MHC-SF data. Among the 551 students in the control group included in this validation study, 541 (98.2%) were followed up and provided complete data at endline.

A total of 657/1082 participants (60.7%) were girls. The mean (standard deviation) age of the participants was 15.3 years (0.3).

### Exploratory factor analysis

The scree plot of the exploratory factor analysis of the CSES-V (Additional file 1: Fig. S1) shows that eigenvalues seem to level off between three and four factors, suggesting that the optimal number of factors is three. The three factors with eigenvalues of approximately 1 or higher and together explained 97.6% of the total variance (Additional file 1: Table S1).

There were two items (items 21 and 23) that did not load into any of the three factors after the rotation (Table 1). Items 18 and 22 cross-loaded into two factors and were assigned to Factor 1. Finally, nine items loading into Factor 1 were emotion-focused coping strategies; the 10 items loading into Factor 2 were problem-focused; and the five items loading into Factor 3 were social support/interaction coping strategies.

**Table 1:** Factor loadings from the exploratory factor analysis of the Coping Self-Efficacy Scale-Vietnamese Version (CSES-V)

Number	Item	Factor 1 Emotion-focused	Factor 2 Problem-focused	Factor 3 Social-support
1	Keep from getting down in the dumps	0.61		
2	Talk positively to yourself	0.45		
3	Sort out what can be changed, and what cannot be changed		0.61	
4	Get emotional support from friends and family			0.52
5	Find solutions to your most difficult problems		0.59	
6	Break an upsetting problem down into smaller parts		0.59	
7	Leave options open when things get stressful		0.63	
8	Make a plan of action and follow it when confronted with a problem		0.61	
9	Develop new hobbies or recreations			0.37
10	Take your mind off unpleasant thoughts	0.85		
11	Look for something good in a negative situation	0.54		
12	Keep from feeling sad	0.79		
13	See things from the other person's point of view during a heated argument		0.64	
14	Try other solutions to your problems if your first solutions don’t work		0.68	
15	Stop yourself from being upset by unpleasant thoughts	0.73		
16	Make new friends			0.60
17	Get friends to help you with the things you need			0.73
18	Do something positive for yourself when you are feeling discouraged	0.45		0.35
19	Make unpleasant thoughts go away	0.84		
20	Think about one part of the problem at a time		0.58	
21	Visualize a pleasant activity or place			
22	Keep yourself from feeling lonely	0.49		0.34
23	Pray or meditate			
24	Get emotional support from community organizations or resources			0.55
25	Stand your ground and fight for what you want		0.38	
26	Resist the impulse to act hastily when under pressure		0.36	

Blanks represent factor loading < 0.3

### Measurement invariance

The MGCFA of the three-factor models (Table 2) supported all three levels of measurement invariance between sexes and longitudinal measurement invariance between baseline and endline. The overall MGCFA (the configural models) models fitted the data well and the fitting indices of the metric and scalar models were almost identical to those of the configural models.

**Table 2:** Model fit indices for multiple-group three-factor models testing measurement invariance between male and female participants and longitudinal measurement invariance of the Coping Self-Efficacy Scale-Vietnamese Version (CSES-V)

Model	CFI	TLI	RMSEA	P-value RMSEA < = 0.05	ΔCFI	ΔRMSEA
*Between sexes*						
1.1 Configural	0.968	0.959	0.033	1.00	N/A	N/A
1.2 Metric	0.967	0.96	0.033	1.00	− 0.001	0
1.3 Scalar	0.966	0.961	0.033	1.00	− 0.001	0
*Longitudinal*						
2.1 Configural	0.965	0.955	0.037	1.00	N/A	N/A
2.2 Metric	0.964	0.956	0.037	1.00	− 0.001	0
2.3 Scalar	0.964	0.957	0.036	1.00	0	− 0.001

CFI: Comparative Fit Index; TLI: Tucker–Lewis index; RMSEA: Root Mean Square Error of Approximation; ΔCFI: CFI difference to previous model; ΔRMSEA: RMSEA difference to previous model; N/A: not applicable

### Correlations and internal consistency

The three factors of the CSES-V were correlated with each other at moderate levels (Table 3), which supports that the three factors are different facets of the same construct, namely coping self-efficacy. All three factors were positively associated with the MHC-SF and negatively associated with the DASS21 sub-scales at a low or moderate level, as hypothesised.

**Table 3:** Correlations between the Coping Self-Efficacy Scale-Vietnamese Version (CSES-V) and mental health scales

	Factor 1 Emotion-focused	Factor 2 Problem-focused	Factor 3 Social-support
Factor 1 Emotion-focused	1		
Factor 2 Problem-focused	0.70	1	
Factor 3 Social-support	0.60	0.60	1
MHC-SF	0.52	0.56	0.57
DASS21-D	− 0.38	− 0.54	− 0.46
DASS21-A	− 0.28	− 0.39	− 0.30
DASS21-S	− 0.29	− 0.48	− 0.32

MHC-SF: Mental Health Continuum Short Form; DASS21-D: Depression Anxiety and Stress Scales 21 items-Depression subscale; DASS21-A: Depression Anxiety and Stress Scales 21 items-Anxiety; DASS21-S: Depression Anxiety and Stress Scales 21 items-Stress

Cronbach’s alpha coefficients of the factors 1, 2, 3 and the whole CSES-V were at acceptable levels (0.91, 0.86, 0.75, and 0.93, respectively).

## Discussion

This study established the evidence of the construct validity of the CSES-V for use among adolescents in Vietnam. The findings strongly confirm the factorial structure of the CSES-V with three factors. All levels of measurement invariance between males and females and longitudinal measurement invariance were strongly supported. All three factors were found to have acceptable internal consistency and were correlated with several mental health measures, as expected.

Like the original validation study of the CSES [7], this study found the same three-factor structure: emotion-focused, problem-focused, and social support/interaction coping strategies. We suggested the exclusion of items 21 ‘Visualize a pleasant activity or place’ and 23 ‘Pray or meditate’ as they had factor loadings lower than the cut-off. These items also had lowest factor loadings among the items loaded into the problem-focused factor in the original validation study [7]. Positive imagery is a technique commonly used in psychotherapy for stress reduction [55]. It might be not commonly used among general population, including adolescents, because some guidance and practice may be needed in order to integrate it as an individual coping strategy. ‘Pray or meditate’ was also one of three items suggested for exclusion by Colodro et al.’s study in the general population in the UK [18], but this item had a good correlation with the total scale score in a study in Iran [19]. ‘Pray or meditate’ is a coping strategy that may be more commonly used by people who are spiritual or who practice meditation. In Vietnam, Buddhism has historically been the dominant religion. However, nowadays many people, especially adolescents, are not showing as much commitment to religious beliefs. This may explain why ‘pray or meditate’ was not a strategy widely endorsed among Vietnamese adolescents.

There are a few inconsistencies between the findings of this study and the original validation study of the CSES [7]. Item 2 ‘Talk positively to yourself’ loaded into the problem-focused factor and item 18 ‘Do something positive for yourself when you are feeling discouraged’ loaded into social support factor in the original validation study, but both loaded into the emotion-focused factor in our study. Problem-focused coping strategies concentrates on changing the stressor itself while emotion-focused coping centres around managing emotional responses to stressful events [1]. ‘Talk positively to yourself’ cannot directly modify the stressor itself but it is a regulative effort to diminish the emotional consequences of stressful events. Therefore, item 2 is more relevant to emotion regulation than problem-focused strategies. ‘Do something positive for yourself when you are feeling discouraged’ can be related to social support strategies if the individual gets support from friends and/or families that is also positive for themselves. However, in the data of this study, this item is more relevant to emotion-focused strategies and it makes sense as ‘do something positive for yourself’ can directly improve their emotional status.

Item 9 ‘Develop new hobbies or recreations’ and item 24 ‘Get emotional support from community organisations or resources’ had low factor loadings in the original validation study which only included adults, but acceptable factor loadings in our study. These results suggest that these two items may be more relevant to adolescents than to adults. It is known that the ability to learn new things peaks in early childhood and adolescence and reduces gradually in adulthood [56]. Therefore, adolescents might be more likely than adults to develop new hobbies or skills to respond to stressful events or situations. Adolescents are often still dependent on their parents/carers, and thus may be accustomed to seeking help from their immediate family, or being supported by their parents/carers to seek external support. In contrast, adults are more often living independently, and may therefore have less support, or find it more challenging to seek help from services or community organisations.

This is, to our knowledge, the first attempt to validate a coping self-efficacy measure for use among adolescents. We provide evidence on multiple aspects of the construct validity using a large sample size. However, we acknowledge several methodological limitations of this study. First, we included adolescents attending school in Hanoi and in a narrow age range. This specific sample may affect generalisation of the findings to the all Vietnamese adolescents. Criterion validity (how well the scale scores agree with a ‘gold standard’ measure), which is an important aspect of construct validity, was not evaluated in this study. We were not able to find a gold standard measure of coping self-efficacy.

### Implications and conclusions

The evidence of the construct validity of the CSES-V in Vietnamese adolescents is established. This scale may be useful for school counsellors or clinical psychologists who work with adolescents, school mental health programs, primary health care, and research on adolescents’ stress and coping. We recommend that the continuous scores of this scale are used rather than any categories, because no cut-off points have been validated to date.

## Supplementary Information

**Additional file 1**. **Fig. 1.** Scree plot - Exploratory factor analysis of the Coping Self-efficacy Scale – Vietnamese Version (CSES-V). **Table 1.** Exploratory factor analysis of Coping Self-efficacy Scale – Vietnamese Version (CSES-V).

## Data Availability

The data, analytic methods (code) used in the analysis, and materials used to conduct the research will be made available to any researcher for purposes of reproducing the results or replicating the procedure on reasonable request to the corresponding author.

## Abbreviations

CSES: Coping Self-efficacy Scale
CFI: Comparative Fit Index
DASS21-S: Depression Anxiety and Stress Scales 21 items
DASS21-D: Depression Anxiety and Stress Scales 21 items-Depression subscale
DASS21-A: Depression Anxiety and Stress Scales 21 items-Anxiety subscale
DASS21-S: Depression Anxiety and Stress Scales 21 items-Stress subscale
MGCFA: Multiple group confirmatory factor analysis
MHC-SF: Mental Health Continuum Short Form
RMSEA: Root Mean Square Error of Approximation
TLI: Tucker–Lewis index
