# Supplementary material for: Validation of the coping self-efficacy scale: Vietnamese version for adolescents
Source: BMC Psychol. 2022 Mar 9;10:59. doi: 10.1186/s40359-022-00770-3 (PMC8905823; doi:10.1186/s40359-022-00770-3)
Supplement: Supplementary file 1 — Additional file 1. Fig. 1. Scree plot - Exploratory factor analysis of the Coping Self-efficacy Scale – Vietnamese Version (CSES-V). Table 1. Exploratory factor analysis of Coping Self-efficacy Scale – Vietnamese Version (CSES-V). [file 40359_2022_770_MOESM1_ESM.docx]

Additional File 1

Fig. 1 Scree plot - Exploratory factor analysis of the Coping Self-efficacy Scale – Vietnamese Version (CSES-V)

Table 1 Exploratory factor analysis of Coping Self-efficacy Scale – Vietnamese Version (CSES-V)

|  | Eigenvalue | Total percent variance explained (%) |
| --- | --- | --- |
| Factor 1 | 9.46 | 80.0 |
| Factor 2 | 1.16 | 9.8 |
| Factor 3 | 0.91 | 7.7 |
| Factor 4 | 0.43 | 3.6 |
| Factor 5 | 0.39 | 3.3 |
